# Reliability of the Single-Leg, Medial Countermovement Jump in Youth Ice Hockey Players

**DOI:** 10.3390/sports9050064

**Published:** 2021-05-13

**Authors:** Anthony S. Donskov, Jeffrey S. Brooks, James P. Dickey

**Affiliations:** 1 Department of Kinesiology, University of Western Ontario, London, ON N6A 357, Canada; jbrook45@uwo.ca (J.S.B.); jdickey@uwo.ca (J.P.D.); 2 Donskov Strength and Conditioning, 7061 Huntley Rd, Columbus, OH 43229, USA

**Keywords:** youth ice hockey, force plates, reliability

## Abstract

Appropriate performance tests are critical for documenting training, fatigue and injury-related changes. Functional performance testing can provide quantitative information on specialized sport movements. The single-leg, medial countermovement jump is an objective measure of frontal plane force, velocity and power, and is particularly applicable for ice hockey players given that ice skating involves applying lateral forces. This study assessed the short-term reliability (10 days) of the single-leg, medial countermovement jump performed by ten competitive male youth ice hockey players. Each participant performed three right and three left maximal single-leg, medial countermovement jumps from force plates. Measured variables included lateral and vertical takeoff velocity, lateral and vertical maximal force, maximal force above bodyweight, lateral and vertical peak concentric power, average concentric power, and average concentric power during the last 100 ms of push-off. Relative reliability was quantified by intraclass correlations. Absolute reliability and the smallest real difference were also calculated. The single-leg, medial countermovement jump had moderate-to-excellent test–retest reliability (ICC: 0.50–0.98) for all twelve variables of interest. These results suggest that the single-leg, medial countermovement jump is a reliable test for assessing frontal plane force, velocity and power in ice hockey players, and is a valid functional performance test for this population given the similarity to ice skating.

## 1. Introduction

Approximately one half of National Hockey League (NHL) players will experience an injury during the course of the season resulting in a loss of playing time. During the 2009–2010 and 2011–2012 seasons, researchers observed that injuries represented a total salary cost of $218 million per year for the NHL teams and their insurance companies [1]. The lower extremity was the most commonly injured area of the body, accounting for 30% of total annual lost salary [1]. The risk of injury is also a concern at the youth level, where lower extremity injuries account for approximately 20–40% of all injuries [2,3,4]. More than 50% of injuries in boys’ ice hockey result in a minimum of one week of lost play [4]. Injury prevention programs must account for numerous physical qualities such as flexibility, power, strength and endurance in order to return players back to sport safely [5,6]. Functional performance tests have been used to assess physical qualities and determine rehabilitation timelines [7,8]. However, biomechanical and reliability considerations need to be examined prior to choosing each test.

Skating is an essential skill for ice hockey players. The authors will be referring to ice hockey when stating hockey for the remainder of the manuscript. The ice surface has a low coefficient of friction [9], precluding force along the skate blade [10]. Accordingly, propulsive force is created on the ice by pushing laterally with the foot [11]. On-ice propulsion involves frontal plane forces, which differs from sprinting on land. Sprinting on land involves force generation predominantly in the sagittal plane [12]. Propulsion occurs by pushing down and into the ground. Accordingly, the differences between the biomechanics of skating and sprinting indicate that these activities should have different performance tests.

Performance professionals use functional performance tests, such as the vertical jump, to assess performance and to guide the rehabilitation process [13,14]. However, the best tests for assessing readiness to return to sport are those that closely mimic the biomechanics of the sporting activity [5]. Vertical jumps and skating involve different push-off mechanics [15]. In both activities, the center of gravity is accelerated by the push-off force. However, push-off force in skating occurs by pushing laterally on the ice. Skaters rely on the reactive force that is perpendicular to the skate blade [16]. Skaters propel forward by external hip rotation, ankle and blade pronation and applying lateral force. These skating mechanics are not incorporated in the standard vertical jump. However, the single-leg, medial countermovement jump provides similar push-off mechanics as experienced in skating.

The reliability of kinetic and temporal variables in the single-leg, medial countermovement jump has been investigated and deemed reliable for field and court sport athletes [17], but not for hockey players. Establishing reliability of force and power variables in the single-leg, medial countermovement jump will support its use as a testing, training and rehabilitation tool used for youth hockey players. Accordingly, the primary purpose of this study was to determine the short-term reliability of the parameters involved in the single-leg, medial countermovement jump. We hypothesized that the single-leg, medial countermovement jump would be a reliable test in male youth hockey players.

## 2. Materials and Methods

### 2.1. Subjects and Study Design

A power analysis identified that ten participants provide 80% power to detect an intraclass correlation coefficient (ICC) of 0.7 at *p* = 0.05 [18]. Ten youth male ice hockey players from a 16U hockey team (16.10 ± 0.32 years old, 181.40 ± 5.38 cm, 78.76 ± 12.81 kg) playing in the Tier 1 AAA Elite Hockey League participated in this study. Participants represented all playing positions (forward, defense, goaltender). All participants had medical clearance from a healthcare professional to participate in this study and self-declared that they were free of any lower body musculoskeletal injuries. Inclusion criteria included no pre-existing medical conditions and currently participating in organized hockey. All participants received written explanation of the study and oral explanation of each test. The Western University Health Science Research Ethics Board approved the experimental protocol (protocol 113858). 

### 2.2. Procedures

The participants were tested on two separate occasions, ten days apart. Testing took place indoors at the Donskov Strength and Conditioning training facility (Columbus, OH, USA), and at a hockey rink (Ice Haus, Columbus, OH, USA). Testing times, warm-ups and jump randomization were identical for both testing sessions. No familiarization trials were performed prior to collecting the single-leg, medial countermovement jumping trials; however, all participants were familiar with these jumps as they were part of their weekly in-season strength and conditioning sessions. 

Participants completed a general warm-up consisting of 15 minutes of static stretching, mobility and dynamic movement (foam rolling, knee hugs, heel to butt, reverse lunge, single-leg deadlift with reach, “A” skips, back pedaling, short accelerations). All participants adhered to the standardized testing instructions. Participants performed three repetitions each, of both left and right single-leg, medial countermovement jumps. Jumps were performed in blocks for each direction, and the order of each block was randomized. Ground reaction forces during the jumps were measured using bilateral force plates (OR6-7, AMTI, Watertown, MA, USA). The force plate signals were sampled at 200 Hz with a 16 bit analog-to-digital converter (USB 6211, National Instruments, Austin, TX, USA) using a custom LabVIEW program (LabVIEW 2012, National Instruments, Austin, TX, USA). One minute of rest was provided between jumps to prevent fatigue [19].

### 2.3. Jump Protocol

All jump trials were administered by the same researcher using standardized verbal commands and demonstrations. Players were instructed to achieve the greatest vertical and horizontal displacement during each jump. Verbal encouragement was offered by the coaching staff to ensure maximal effort on each attempt. Compromised trials (improper technique, equipment malfunction) were discarded and repeated [20]. During the single-leg, medial countermovement jump, participants started standing with one foot on either force plate and then stood on the designated leg, squatted to a self-selected depth, and then jumped medially as far as possible to land on both legs on the ground. Arm swing was permitted. Two strength and conditioning professionals monitored all jumps to ensure proper jumping technique and safe landing mechanics. 

### 2.4. Data Processing 

**Figure 1:**
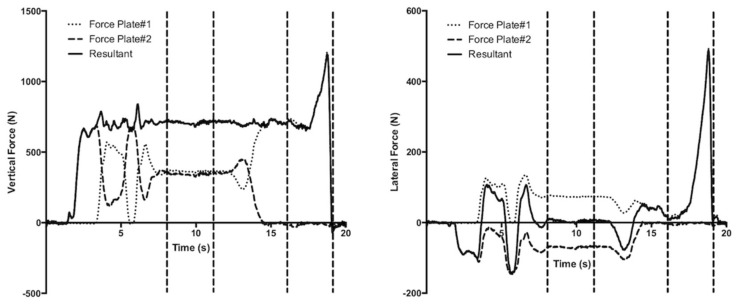
Vertical (**left** panel) and lateral (**right** panel) forces during a single-leg, medial countermovement jump from a representative trial. The three traces represent force plate #1 (dotted line), force plate #2 (dashed line) and resultant force (solid line). From left to right on each panel, the four vertical dashed lines reflect the beginning and end of standing prior to the jump, the initiation of jump (lateral forces >10 N) and end of jump.

All data analysis was performed using custom software in LabVIEW. We did not filter the force signals. The forces in the X, Y and Z directions were summed from each force plate to capture the forces applied through each limb, and to represent the total ground reaction force acting on the participant. The average of the three jump trials was used for analysis. Bodyweight was collected from standing trials. Jump phases were determined using an automated procedure, similar to previous research [21,22], and were verified using visual inspection. The initiation of the jump was defined as the point where lateral force increased 10 N above baseline. The end of the propulsive phase was defined as the point where the force dropped to less than 10 N. The start of the concentric phase for both vertical and lateral forces and accelerations was determined when the velocity of the center of mass became positive for more than 0.1 consecutive seconds (Figure 1). The net vertical impulse was calculated by subtracting the gravitational impulse from the total impulse. Vertical and lateral take off velocities were calculated using the impulse momentum relationship. Vertical and lateral power were calculated as the product of velocity and force. Maximum force was extracted from the force-time curves. Peak concentric power, average concentric power and average concentric power in the last 100 ms were extracted from the power curves. 

Force was expressed in N (i.e., not normalized to body weight) and power was expressed in W (i.e., not normalized). Force was also expressed relative to body weight, for both lateral and vertical maximal forces.

### 2.5. Statistical Analysis

A correlation matrix was created in order to identify the relationships among variables in the single-leg, medial countermovement jump. This was based on pooled data from both test sessions. The size of the correlation was evaluated as follows: *r* < 0.7 low; 0.7 ≤ *r* < 0.9 moderate, and *r ≥* 0.9 high [23]. Coefficients of determination (r^2^) were calculated to indicate the percent of common variance explained by the correlation [24]. Shapiro–Wilk tests were used to assess data normality [25]. Normal data are presented as mean ± one standard deviation (SD). 

Reliability analyses were performed using the Hopkins spreadsheet [26]. A variety of reliability calculations were used as there is no gold standard for this test [27,28]. A two-way random-effects model ICC (3,1) was used to evaluate relative reliability [27]. Values less than 0.5, between 0.5 and 0.75, between 0.75 and 0.9, and greater than 0.9 were interpreted as poor, moderate, good, and excellent reliability, respectively [29]. The standard error of measurement (SEM) was used to assess absolute reliability [30]. Relative SEM (SEM%) was quantified by dividing the SEM by the mean of all the data from the two test occasions. The smallest real difference (SRD) was calculated by multiplying the SEM by 1.96 and by the square root of 2.0 to include 95% of the observations of the difference between the two measurements [30]. The normalized SRD, expressed as a percentage (SRD%), was calculated by dividing the raw SRD by the mean of all the data from the two test occasions.

## 3. Results

Normality was confirmed (*p* > 0.05) for all variables. The strength of the relationships between variables are presented in the correlation matrix (Table 1). There was a high correlation between vertical concentric average power in the last 100 ms and vertical peak concentric power (*r* = 0.99). In addition, a near-perfect relationship was observed for lateral concentric average power in the last 100 ms and lateral peak concentric power (*r* = 0.99). Several notable relationships were observed among the variables including a moderate relationship between maximum vertical force and peak lateral concentric power (*r* = 0.72), an inverse relationship between vertical takeoff velocity and lateral takeoff velocity (*r* = −0.36), and a large degree of independence between average and peak concentric power for both the lateral and vertical directions (*r* = 0.58; *r^2^* = 0.33 and *r* = 0.63; *r*^2^ = 0.40, respectively).

The reliability of the variables of interest are presented in Table 2. We observed moderate-to-excellent reliability for all twelve variables of interest (ICCs between 0.50 and 0.98) for both right and left jumps. Excellent reliability for both right and left leg jumps was observed for maximum lateral force, maximum vertical force, and vertical average concentric power in the last 100 ms (ICCs > 0.91). The SRD%s ranged from 5.2 to 6.5% for maximum vertical force for both left and right legs, to 14.8 to 16.5% for vertical average concentric power during the last 100 ms for both the left and right legs. The SEM%s also ranged for each variable with maximum vertical force at 1.9% and 2.3% for the left and right legs to 5.35% and 5.9% for vertical average concentric power during the last 100 ms for both the left and right legs.

We observed good reliability for lateral takeoff velocity, maximal vertical force above body weight (%BW), lateral peak concentric power, lateral average concentric power during the last 100 ms, and vertical peak concentric power for both right and left jumps (ICCs between 0.75 and 0.98). The SRD%s ranged from 6.7% to 14% for lateral takeoff velocity for both the right and left legs, to 17.4% and 21% for vertical peak concentric power for both the right and left legs. The SEM%s ranged from 2.2 to 4.9% for lateral takeoff velocity on the right and left legs to 6.2 to 7.7% for peak concentric power on the right and left legs, respectively.

Moderate reliability was observed for maximum lateral force above body weight (%BW), vertical takeoff velocity, lateral average concentric power and vertical average concentric power in both legs. Minimal differences were found between trial one and two for vertical average concentric power (710.66 ± 225.30 W vs. 686.18 ± 199.27 W right leg, 607.04 ± 131.84 vs. 638.30 ± 189.26 W left leg). Inter-limb reliability differences were observed for each parameter; however, all parameters met the moderate-to-excellent rating. The right limb sustained higher reliability in all lateral force, velocity and power parameters, while the left limb sustained higher reliability during all vertical force, velocity and power parameters with the exception of vertical average concentric power.

## 4. Discussion

The primary purpose of this study was to determine the short-term reliability of the parameters involved in the single-leg, medial countermovement jump. We hypothesized that the single-leg, medial countermovement jump would be a reliable test in male youth hockey players. Our hypothesis was supported for all twelve discrete variables.

Research in field-based sports has concluded that the medial countermovement jump can be used to reliably measure force and power in the frontal plane [17,31]. The current research extends this finding by demonstrating that it is reliable in measuring force and power in youth hockey players. Given biomechanical similarities between this jump and the propulsion phase in skating, it is likely that this jump is an important off-ice test to evaluate skating performance. To our knowledge, this paper is the first to present SEMs and relative SEMs for the various jump variables associated with the single-leg, medial countermovement jump. These numbers serve as baseline measures for future research.

Other metrics have been used to assess skating performance including the vertical jump, squat jump, forty-yard dash, thirty-meter test, broad jump, and the triple hop jump test [32,33,34]. Studies vary in concluding which test most accurately assesses on-ice skating performance. One study observed that vertical jump impulse, as measured on force plates, was one of several variables that best assessed on-ice skating performance [32], while others have stated that the thirty-meter sprint and triple hop were superior [33]. Finally, in determining the measurement device and jumping protocol most appropriate for testing elite hockey players, one study concluded that the Vertec squat jump was superior to the Just Jump mat for measuring lower body power [35]. True countermovement is uncommon in ice hockey as players rarely use the stretch-shortening cycle to enhance muscle contraction [31]. In addition, push-off on the ice is different than on land. Hockey players must push off laterally in order to create propulsive force. This push-off is similar to the single-leg, medial countermovement jump. Since the best tests for assessing readiness to return to sport are those that closely mimic the biomechanics of the sporting activity [5], the single-leg, medial countermovement jump appears to be an excellent functional performance test for skating.

Other studies have measured the reliability of the single-leg, medial countermovement jump. Measuring distance jumped showed a pooled ICC of 0.97 for both men and women with intrasubject variability, expressed as a coefficient of variation, of 4.6% [36]. However, the use of measures such as distance jumped does not measure ground reaction forces. Individuals recovering from lower-extremity injuries employ unique jumping strategies that may not present when measuring distance jumped. While injured, an athlete may select a movement strategy that avoids force application to the injured limb [33]. Therefore, the use of force plates to measure ground reaction forces and leg asymmetries is critical for both healthy and injured athletes [37]. Vertical and lateral ground reaction forces during the single-leg, medial countermovement jump were investigated for field and court sport athletes [17]. For the concentric variables, peak vertical force (ICC = 0.96), peak lateral ground reaction (ICC = 0.89) and peak vertical power (ICC= 0.86) were reliable measures. Similarly, our investigation observed ICCs ranging from 0.88 to 0.98 for these variables. The single-leg, medial countermovement jump has also differentiated between elite and non-elite soccer players [31]. Researchers observed that single-leg jumps such as the unilateral vertical jump, unilateral horizontal jump and unilateral medial countermovement jump could differentiate elite from non-elite soccer players and therefore should be included in power profiling assessments.

Lateral push-off, during which forces are produced perpendicular to the skate blade, occurs in a short window of time [15]. Our results suggest that this can be measured using the medial countermovement jump. Mean average lateral concentric power during the last 100 ms prior to push-off showed good-to-excellent reliability (ICC = 0.86–0.98). In addition, the single-leg, medial countermovement jump displayed larger horizontal takeoff velocities than vertical takeoff velocities. Vertical takeoff velocity was 61% of total lateral takeoff velocity during right and left leg propulsion. This suggests that larger horizontal forces were needed to move the center of mass effectively during this jump, solidifying its use for measuring hockey player performance.

From an injury-risk perspective, the single-leg, medial countermovement jump may be useful for measuring force, velocity and power of the lower limbs prior to potential injury occurrence. This provides objective information to the performance staff and may serve to guide rehabilitation during return to play. In addition, the single-leg, medial countermovement jump may be used to assess and track asymmetries between right and left limbs in both healthy and injured athletes. It has been noted that interlimb differences of greater than ten percent lead to a fourfold increase in re-rupture of the ACL in athletes [38]. Having a reliable, frontal plane test that can provide information pertaining to jump performance may be used to improve return-to-play procedures in hockey.

A number of features of the single-leg, medial countermovement jump are similar to skating. For example, it has a high concentric effort, minimal stretch-shortening cycle, arm swing to assist propulsion, and frontal plane force production. These features of the single-leg, medial countermovement jump substantiate its construct validity [25] as an assessment of skating propulsion. Accordingly, it is an important test for ice hockey players.

### Limitations

There are limitations to this study. This study tested a narrow age range of youth athletes playing in a single youth hockey league. Future research should evaluate this jump with a broader age range of hockey players. We included players of different playing positions, which may have affected results. Further research should evaluate whether there are systemic differences in single-leg, medial countermovement jump performance between player positions. We did not ask our participants about limb dominance, and therefore are unable to evaluate whether the bilateral differences in reliability may be due to limb dominance. Lastly, the location of testing may have affected performance. Testing took place both at the gym and at the rink. Different locations and temperatures may have altered physiologic behavior, causing a potential change in performance.

## 5. Conclusions

All twelve discrete variables examined showed moderate-to-excellent between-session reliability. Specifically, both lateral and vertical ground reaction forces showed the highest reliability. Lateral takeoff velocity and lateral average concentric power during the last 100 ms showed good reliability. As a result, performance professionals can feel confident using these variables extracted from single-leg, medial countermovement jumps to gauge hockey player performance.

In conclusion, the results from this study suggest that the single-leg, medial countermovement jump is a reliable test of frontal plane force production for youth hockey players. The fact that the single-leg, medial countermovement jump allows the tester to measure single-leg ground reaction forces and power in the frontal plane makes this test a relevant option in all phases of sport performance and rehabilitation. A larger sample size including athletes of different ages is needed to evaluate changes with training and recovery from injury for ice hockey players.

## Figures and Tables

**Table 1:** Correlation Matrix for the parameters derived from the single-leg, medial countermovement jump.

Parameter	VERT VTO (m/s)	VERT Jump Height (cm)	VERT Peak Concentric Power (W)	VERT Average Concentric Power (W)	VERT Average Concentric Power 100 ms (W)	LAT VTO (m/s)	LAT Peak Concentric Power (W)	LAT Average Concentric Power (W)	LAT Average Concentric Power 100 ms (W)	Maximum VERT Force (N)	Maximum VERT Force above Body Weight (%BW)	Maximum LAT Force (N)
VERT jump height (cm)	1.00	-	-	-	-	-	-	-	-	-	-	-
VERT peak con power (W)	0.77	0.77	-	-	-	-	-	-	-	-	-	-
VERT Avg con power (W)	0.55	0.55	0.58	-	-	-	-	-	-	-	-	-
VERT Avg con Power 100 ms (W)	0.76	0.76	1.00	0.58	-	-	-	-	-	-	-	-
LAT VTO (m/s)	−0.36	−0.36	−0.08	−0.09	−0.05	-	-	-	-	-	-	-
LAT peak con power (W)	−0.18	−0.18	0.32	0.13	0.34	0.84	-	-	-	-	-	-
LAT Avg con power (W)	−0.30	−0.30	0.02	0.48	0.03	0.61	0.63	-	-	-	-	-
LAT Avg con power 100 ms (W)	−0.20	−0.20	0.32	0.10	0.34	0.85	1.00	0.63	-	-	-	-
Max VERT force (N)	0.22	0.220	0.77	0.42	0.77	0.30	0.72	0.45	0.72	-	-	-
Max VERT force above body weight (%BW)	0.40	0.40	0.52	0.27	0.50	−0.09	0.13	0.17	0.15	0.55	-	-
Max lateral force (N)	−0.07	−0.07	0.50	0.19	0.51	0.63	0.95	0.54	0.94	0.86	0.26	-
Max LAT force above body weight (%BW)	−0.22	−0.22	0.07	−0.14	0.08	0.70	0.78	0.43	0.77	0.41	0.29	0.73

VERT: vertical; VTO: vertical takeoff velocity; LAT: lateral; LVTO: lateral takeoff velocity; Max: maximum; Con: concentric; %BW: percent bodyweight. Correlation coefficient magnitudes larger than 0.3125 are statistically significant at *p* < 0.05.

**Table 2:** Test–retest reliability for the parameters involved in the single-leg, medial countermovement jump.

Single-Leg, Medial Countermovement Jump Force and Velocity Variables						
*Lateral Force/Velocity*	Mean (SD) Trial 1	Mean (SD) Trial 2	SEM	Typical Error (90% CI)	SRD	ICC (90% CI)
(R) Max LAT Force (N)	487.0 (87.0)	510.2 (85.4)	11.0	12.8 (9.4–21.2)	30.6	0.98 (0.95–0.99)
(L) Max LAT Force (N)	504.1 (79.0)	502.2 (83.5)	23.6	27.8 (20.8–45.8)	65.4	0.91 (0.74–0.97)
(R) Max LAT force above body weight (%BW)	65.1 (4.9)	68.0 (4.8)	1.9	2.1 (1.5–3.5)	5.2	0.85 (0.59–0.950
(L) Max LAT force above body weight (%BW)	67.6 (5.4)	67.0 (5.8)	3.5	3.8 (2.8–6.3)	9.6	0.59 (0.10–0.85)
(R) LAT VTO (m/s)	2.24 (0.18)	2.18 (0.15)	0.05	0.05 (0.04–0.09)	0.15	0.91 (0.76–0.97)
(L) LAT VTO (m/s)	2.22 (0.22)	2.20 (0.18)	0.11	0.11 (0.08–0.18)	0.31	0.75 (0.38–0.91)
*Vertical Force/Velocity*						
(R) Max VERT Force (N)	1272 (181.2)	1273 (190.8)	29.6	35.6 (26.0–58.6)	82.1	0.97 (0.92–0.99)
(L) Max VERT Force (N)	1304 (200.7)	1261 (188.6)	24.4	27.9 (20.4–46.0)	67.9	0.98 (0.95–1.00)
(R) Max VERT force above body weight (%BW)	70.8 (11.4)	70.1 (10.9)	4.6	4.9 (3.6–8.2)	12.6	0.84 (0.57–0.95)
(L) Max VERT force above body weight (%BW)	74.8 (11.7)	68.3 (9.4)	3.5	3.6 (2.6–6.0)	9.7	0.91 (0.74–0.97)
(R) VERT VTO (m/s)	1.38 (0.16)	1.35 (0.08)	0.12	0.09 (0.07–0.16)	0.32	0.50 (−0.03–0.81)
(L) VERT VTO (m/s)	1.37 (0.27)	1.30 (0.27)	0.10	0.11 (0.08–0.19)	0.29	0.85 (0.59–0.95)
**Single-Leg, Medial Countermovement Jump Power Variables**						
*Lateral Power*	Mean (SD) Trial1	Mean (SD) Trial2	SEM	Typical Error (90% CI)	SRD	ICC (90% CI)
(R) LAT peak con power (W)	925.1 (225.2)	945.3 (209.2)	38.6	43.6 (31.8–71.7)	107.1	0.97 (0.91–0.99)
(L) LAT peak con power (W)	934.9 (204.1)	930.8 (206.7)	84.8	95.9 (67.0–157.8)	235.1	0.82 (0.54–0.94)
(R) LAT Avg con power (W)	406.6 (135.8)	370.4 (117.5)	53.9	56.6 (41.3–93.2)	149.4	0.84 (0.57–0.95)
(L) LAT Avg con power (W)	377.3 (164.7)	378.6 (150.9)	90.5	94.2 (68.7–155.0)	250.8	0.70 (0.28–0.89)
(R) LAT Avg con power (100 ms; W)	873.1 (221.8)	887.7 (207.0)	28.0	31.9 (23.2–52.4)	77.7	0.98 (0.95–0.99)
(L) LAT Avg con power (100 ms; W)	886.9 (199.2)	879.9 (193.1)	73.8	82.0 (59.8–134.9)	204.6	0.86 (0.62–0.95)
*Vertical Power*						
(R) VERT peak con power (W)	1663.5 (306.7)	1668 (254.8)	104.6	109.2 (79.7–179.8)	290.2	0.88 (0.67–0.96)
(L) VERT peak con power (W)	1690.4 (458.5)	1591 (382.9)	127.2	134.7 (98.3–221.7)	352.7	0.92 (0.77–0.98)
(R) VERT Avg con power (W)	710.6 (225.3)	686.1 (199.2)	84.6	90.1 (65.7–148.3)	234.7	0.85 (0.61–0.95)
(L) VERT Avg con power (W)	607.0 (131.8)	638.3 (189.2)	70.0	94.4 (68.9–155.4)	194.2	0.72 (0.31–0.90)
(R) VERT Avg con power 100 ms (W)	1582.1 (310.1)	1616 (256.2)	95.2	99.8 (72.8–164.3)	263.9	0.91 (0.73–0.97)
(L) VERT Avg con power 100 ms (W)	1621.0 (445.0)	1556 (368.1)	128.4	135.2 (98.6–222.4)	356.0	0.92 (0.76–0.97)

R: right leg; L: left leg; VERT: vertical; LAT: lateral; Avg: average; con: concentric; SD: standard deviation; SEM: standard error of measure; SRD: smallest real difference; CI: confidence interval; LAT VTO: lateral takeoff velocity; VERT VTO: vertical takeoff velocity; %BW: percent bodyweight; ICC: intraclass coefficient.

## Data Availability

The data presented in this study are available upon request from the corresponding author.
